# Computational Methods and Software Tools for Functional Analysis of miRNA Data

**DOI:** 10.3390/biom10091252

**Published:** 2020-08-28

**Authors:** Adrian Garcia-Moreno, Pedro Carmona-Saez

**Affiliations:** 1 Bioinformatics Unit, Centre for Genomics and Oncological Research (GENyO)—Pfizer/University of Granada/Andalusian Regional Government, PTS Granada, 18016 Granada, Spain; adrian.garcia@genyo.es; 2 Department of Statistics, University of Granada, 18071 Granada, Spain

**Keywords:** functional analysis, miRNA, ncRNA, databases, enrichment, tools

## Abstract

miRNAs are important regulators of gene expression that play a key role in many biological processes. High-throughput techniques allow researchers to discover and characterize large sets of miRNAs, and enrichment analysis tools are becoming increasingly important in decoding which miRNAs are implicated in biological processes. Enrichment analysis of miRNA targets is the standard technique for functional analysis, but this approach carries limitations and bias; alternatives are currently being proposed, based on direct and curated annotations. In this review, we describe the two workflows of miRNAs enrichment analysis, based on target gene or miRNA annotations, highlighting statistical tests, software tools, up-to-date databases, and functional annotations resources in the study of metazoan miRNAs.

## 1. Introduction

Since the discovery of interfering RNAs in 1993 in *Caenorhabditis elegans* [1] miRNAs have been continuously characterized by high throughput experimental techniques. miRNAs are non-coding RNA molecules of ~22 nucleotides that mediate gene silencing by guiding Argonaute (AGO) proteins to target sites in the 3′ untranslated region (UTR) of mRNAs. Over the past few decades, more than 2000 miRNAs were discovered in humans [2], and their key roles in many development and biological processes were characterized. miRNAs were also studied in different human diseases and are currently being pursued in clinical diagnostics and as therapeutic targets [3].

There are several resources that store information about miRNAs. miRBase [4] is one of the main databases that contain a complete miRNA catalogue with sequence and functional information covering more than 271 organisms, including 38,589 hairpin precursors and 48,860 mature miRNAs. Other important miRNAs databases are miRCarta [5] and mirGeneDB [6]. MiRCarta contains miRNA and precursor data from miRBase, predicted miRNAs from sequencing data though miRMaster [7] and different publications. mirGeneDB encloses information regarding curated miRNAs across the metazoan phylum. These are invaluable resources, but important considerations must be kept in mind related to false positives. For example, many entries in mirBase were reported to be fragments of other classes of small RNAs including tRNAs, snoRNAs and rRNA. In an effort to discard human false-positive miRNAs collected in these databases, Alles et al. [2] used small RNA sequencing data from almost 30,000 samples from different sources, such as miRMaster, The Cancer Genome Atlas (TCGA) and National Center for Biotechnology Information (NCBI) Sequence Read Archive (SRA), to report 2300 true human mature miRNAs, of which 1115 are currently annotated in miRBase. Increasing knowledge about the role of miRNAs in human diseases also led to the development of dedicated resources, such as Human miRNADisease Database (HMDD) [8], which contains experimentally validated miRNAs, or miRCancer database [9], which incorporates miRNAs mainly associated with different types of cancers. Similar to HMDD, the Mammal NcRNA-Disease Repository (MNDR) [10] gathers further mammalian organisms, offering a broader catalogue of associations between diverse ncRNAs and diseases. The majority of miRNAs databases are dedicated to animals; nevertheless, several are also dedicated to plants, for example, Plant miRNA Encyclopedia (PmiREN) [11] is a complete, up-to-date catalogue of plant miRNAs that encompass other existing plant databases.

Next-generation sequencing (NGS) and microarray technologies were widely used in the past decade to analyze gene mRNAs and miRNAs at the genome level. In the field of transcriptomics, one common experimental set up is to compare different experimental conditions, i.e., disease versus healthy samples, to define a list of dysregulated miRNAs, which could range from dozens to hundreds. A main challenge in this context, and in the characterization of miRNA networks and pathways, is the drawing of conclusions from these miRNAs lists. This is approached by functional analysis, also referred to as enrichment or over-representation analysis, which generally, involves deciding whether miRNAs are significantly enriched in a specific pathway or biological process which may indicate that the process is associated with the observed phenotype. To perform this analysis, functional annotations can be retrieved from databases, such as Gene Ontology (GO) [12] or Kyoto Encyclopedia of Genes and Genomes (KEGG) [13], and statistical tests can be applied to evaluate which terms are over-represented in the list.

Enrichment analysis is widely used for analyzing gene lists, and functional information and annotation databases are usually centered on genes. To implement these types of methods for miRNAs, a common approach is to retrieve their target genes, which are used to infer processes or pathways in which miRNAs are involved. Alternatively, efforts to centralize the information of a direct association between miRNAs and biological processes are being carried out. These types of databases allow researchers to use curated annotations for miRNAs directly for functional analysis, thereby overcoming some known limitations of indirect miRNA-targets annotations. In this context, Huntley et al. [14,15] used Gene Ontology to annotate miRNAs, taking advantage of probably the most well-settled biological ontology in order to directly annotate miRNAs with their functions. They created the initial guidelines, resulting in 500 mature miRNAs from human, mouse, and rat being associated with nearly 4400 GO terms so far, of which over 3000 are linked with human miRNAs.

In this work, we provide a detailed overview of the functional analysis pipeline in miRNAs, including a revision of annotation databases, statistical tests and a comparative analysis of all available software tools. Further information about quantification methods and downstream miRNAs analyses should be regarded in other reviews. For example, smallRNA-seq aligners are compared by Ziemman et al. [16] and four commonly used miRNA-seq analysis tools are comparatively evaluated with a standard toxicogenomics study design by Bisgin et al. [17].

## 2. Functional Analysis Workflow in miRNAs

**Figure 1:**
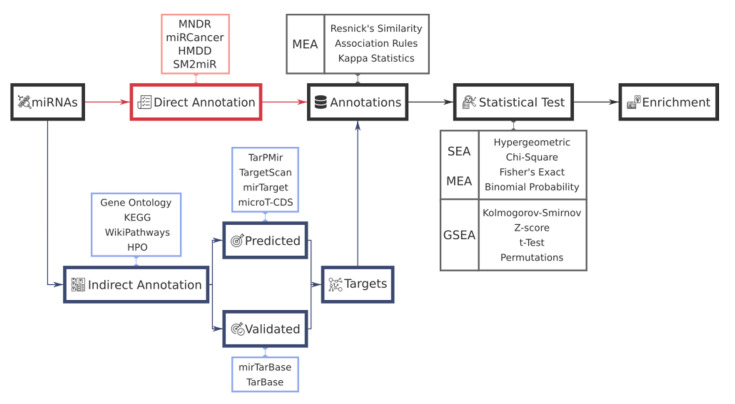
Overview of workflow for functional analysis of miRNAs. Given a list of miRNAs, functional annotations can be retrieved via direct (in **red**) or indirect (in **blue**) schemas. Direct annotations are obtained from dedicated databases (i.e., MNDR, miRCancer, HMDD, SM2miR), in which functional terms are directly associated with miRNAs. In the indirect annotations, schema miRNAs are annotated with terms associated with target genes via gene-centered databases (i.e., Gene Ontology, KEGG, WikiPathways, HPO). Then, miRNAs are transformed to their target genes using prediction algorithms (TarPMir, TargetScan, mirTarget, microT-CDS) or experimentally validated targets databases (mirTarBase, TarBase). Functional terms associated with miRNAs, can be grouped by an MEA approach before statistical analysis. Different statistical tests can be applied, SEA and MEA use the same tests to evaluate the enrichment of annotations in the input list with respect to the reference list. Alternatively, threshold-free-based approaches from GSEA tests can be used to analyze the annotations distribution in the entire ranked list. Finally, p-values assigned to each annotation can be used to define over-represented and significant annotations.

The functional analysis starts with a list of miRNAs (e.g., miRNAs differentially expressed between two phenotypes) and a set of annotations obtained from two different pipelines (see Figure 1). The most common approach is based on obtaining all target genes associated with the list of miRNAs, for which functional gene annotations can be associated, then applying a statistical test to determine their statistical significance; however, there are some reported drawbacks regarding this strategy. Blaezard et al. [18] described that this approach results in certain functional categories being preferentially targeted by miRNAs, regardless of whether those miRNAs are differentially expressed in a biological state or not. This implies that random sets of miRNAs report significant p-values for certain specific terms. Equally, Godard and van Eyll [19] proved that the results are not specific and lead to the systematic identification of highly related biological processes, reporting that random miRNA lists of the same size as the analyzed signature, result in similar enrichment results and demonstrate bias toward cancer and cell cycle terms. Although these are important drawbacks, these analyses are still very useful to derive biological information and are used routinely in published studies. An alternative approach is based on using direct and curated annotations of miRNAs obtained through expert-based annotation from the literature. As mentioned in the introduction, methods following this strategy gained interest in the last few years fueled by the work of Huntley et al. which integrated miRBase and Gene Ontology. Additionally, dedicated databases and enrichment tools using direct annotations are also beginning to be considered, namely, HMDD, MNDR and miRCancer; these databases are described in the following sections.

Once a set of terms associated with miRNAs exists, a statistical test is applied to determine the most representative annotations in the list. Functional analysis can be classified into three different types [20] (1) Singular Enrichment Analysis (SEA), in which each functional term is evaluated individually using different statistical methods, most commonly the hypergeometric distribution (2) Gene Set Enrichment Analysis (GSEA), which also evaluates independent annotations using a whole set of genes/miRNAs as ranked by certain criteria, e.g., fold change, and computes an enrichment score using Kolmogorov-Smirnov statistics; and (3) Modular Enrichment Analysis (MEA), which takes advantages of the inherent relationship among annotations to define sets of terms that are shared by genes/miRNAs to evaluate their significance.

## 3. miRNA-Target Gene Annotation and Resources

Target genes for miRNAs are usually discovered by means of sequence-based prediction algorithms or through experimental validation. In this section, we describe some popular prediction tools and experimentally validated targets databases indicated in Figure 1. We focused on common algorithms used by functional enrichment tools, but more complete reviews on target predictions algorithms are published by Riffo-Campos et al. [21], Peterson et al. [22], and Witkos et al. [23].

### 3.1. miRNA-Target Gene Prediction Methods

miRNA target prediction is mainly performed based on sequence analysis. These type of algorithms take into account several factors, such as seed pairing and sequence similarity, among miRNA and target mRNAs [24], accessibility of an mRNA [25] AU content [26], GU wobble in the seed match [27], 3′ compensatory pairing [28], folding energy [26,29,30], and conservation [31]. Notwithstanding, the binding of a miRNA to its target transcript does not necessarily result in gene expression downregulation. In fact, most observed miRNA binding events, as revealed by crosslinking immunoprecipitation (CLIP) analysis, have little functional consequences [32,33]. Thus, in the context of miRNAs functional analysis, it is important that target prediction algorithms also take into account the effect of target down-regulation by miRNA with RNA-seq data as a method to confirm real functional associations of miRNAs-targets [29]. Table 1 presents a summary of the described prediction tools.

TarPmiR [34], one of the most complete prediction algorithms, introduced several new miRNA-target binding features by applying four different machine learning methods to CLASH (crossinking, ligation and sequencing of hybrids) data. Apart from the previously mentioned conventional features, some novel factors introduced in TarPmiR are the consideration of the m/e motif (how different positions in miRNAs match their corresponding target sites positions), length of the target mRNA region, length of the largest consecutive pairs, the difference between the number of paired positions in the seed region and at the miRNA 3′ end, number of paired positions at the miRNA 3′ end, and position of the largest consecutive pairs relative to the miRNA 5′ end and the total number of paired positions.

Another popular algorithm is TargetScan [32] which is mainly based on seed matching and searches for the presence of conserved 8mer, 7mer, and 6mer sites of mRNA that match each miRNA. Sites with mismatches in the seed region but are compensated by conserved 3′ pairing, and centered sites are also provided. In the last version of TargetScan, predictions of mammal miRNAs are ranked, based on the predicted efficacy of targeting using cumulative weighted context++ scores of the sites. Context++ is a machine learning model resulting from a study of 26 features, 14 of which were included in the algorithm. These features are: (1) 3′UTR target-site abundance, (2) predicted seed-pairing stability, (3) sRNA position 1, (4) sRNA position 8, (5) site position 8, (6) local AU content, (7) 3′ supplementary pairing, (8) predicted structural accessibility, (9) minimum distance, (10) probability of conserved targeting, (11) ORF length, (12) 3′-UTR length, (13) 3′-UTR offset-6mer sites, and (14) open reading frame (ORF) 8mer sites.

MirTarget [35] is the prediction algorithm that gives rise to MirDB [36], which consists of a support vector machine model trained using public RNA-seq data and miRNA-target databases to identify targeting features characteristic of both miRNA binding and target downregulation. Key aspects in this algorithm include seed conservation, seed match specifically in positions 2–8, base composition in the regions flanking the seed pairing sites, secondary structure, site accessibility, free energy, and location of the site within the 3′ UTR. Respectively, MirDB hosts miRNA expression profiles of over 1000 cell lines and presents target prediction data tailored for specific cell models, as well as predictions of miRNA functions by integrative analysis of targets and Gene Ontology data.

DIANA microT-CDS [37] is another algorithm for target prediction, which identifies the most remarkable features extracted from photoactivatable-ribonucleoside-enhanced crosslinking and immunoprecipitation (PAR-CLIP) datasets via machine learning techniques. This results in an algorithm with the ability to discover miRNA whose binding location is directly known in both coding sequences (CDS) and 3′ UTR based on extended seed matching, distance to the nearest end of CDS or 3′ UTR, distance to an adjacent binding site, the free energy of the hybrid, conservation, AU content and 3′ UTR accessibility.

The combination of different target prediction methods is a common approach to get more consistent results. For example, Oliveira et al. [38] concluded that the most effective approach was the union, instead of the intersection, of the results from different algorithms to maximize performance, and that several true targets were not identified by these tools alone. Tabas-Madrid, D. et al. [39] proposed two methods to measure the confidence of predicted interactions based on experimentally validated information. These reassigned new scores and statistical confidences for each predicted interaction by nine studied algorithms. In this way, they reduce the selection of interactions to a unique database based on an intuitive score and allow comparing databases between them. Several authors agree with the union of algorithms methods using different approaches [40,41,42,43].

### 3.2. Validated miRNA-Target Gene Resources

miRTarBase [44] and DIANA-TarBase [45], are two main resources that centralize validated miRNA-target information. An overview of them is presented in Table 2. 

miRTarBase is one of the largest databases of experimentally validated miRNA-target interactions. The last version, published in 2020, contains almost 480,000 validated interactions extracted from manual curation from a corpus of 11,021 articles. This database not only includes targets, but also regulators of miRNAs to investigate the up- and down-regulation of miRNAs. Targets are classified by the experimental technique used in the validation and whether the evidence is weak or strong. Reporter assays, Western blotting, and qRT-PCR qualify as strong evidence, whereas high throughput techniques, e.g., CLIP-seq, microarray, pulsed stable isotope labeling by amino acids in cell culture (pSILAC), etc., are considered weak evidence. Additionally, disease information from HMDD is incorporated into this database.

Within the DIANA suite, TarBase reached its eighth version in 2017, and includes over 670,000 unique miRNA-target pairs. Nearly 1200 manually curated publications and more than 350 high-throughput datasets support miRNAs-target genes evidence in the database. TarBase divides the targets into low- and high-throughput techniques. Some of the most present methods in the database are reporter assays, Western blotting, qPCR, proteomics, biotin miRNA tagging, sequencing data and microarrays, among others. Additionally, disease information from miR2Disease [46] is included.

## 4. Functional Annotation Resources for miRNAs

Functional annotations for miRNAs can be directly assigned by literature curation or inferred using information from target genes. In this section, we provide a brief overview of the main biological information sources used in enrichment analysis for both miRNAs and genes.

### 4.1. miRNA-Based Annotation Resources

Databases that contain functional information of miRNAs compile annotations obtained via manual literature searches or text-mining algorithms plus an expert curation process to check evidence levels. There are many resources focused on associating diseases to miRNAs, while few of them relate them to concrete biological functions or entities, such as transcription factors, drugs, or epigenetic modifiers. We briefly describe some of these databases used in miRNAs enrichment tools.

miRCancer is a reference database which includes human miRNA-cancer associations from empirical evidence; built via text-mining of more than 26,000 PubMed articles, and currently contains 9080 relationships among 57,984 miRNAs and 196 cancers. The algorithm is based on 75 rules, which represent the common sentence structures typically used to state miRNA expressions in cancers. All the annotations are confirmed manually after automatic extraction.

HMDD is focused on establishing human miRNA—disease associations evidence-based on manual curation. Currently, more than 32,281 experimentally supported miRNA—disease links, covering 1102 miRNA genes and 850 diseases from 17,412 papers are contained in the database. All miRNAs included are standardized to match the miRBase nomenclature and diseases are classified and normalized on the basis of Disease Ontology [47] and (Medical Subject Headings) MeSH; associations are categorized in six evidence codes.

In contrast to miRCancer and HMDD, MNDR integrates experimental and predicted ncRNA-disease associations from manual literature curation and 10 other resources for 11 different mammalian organisms. More than one million of ncRNA-disease entries, including 6301 miRNAs, 39,880 lncRNAs, 20,256 circRNAs, 10,894 piRNAs and 521 snoRNAs with over 1600 diseases, are stored in the database. Diseases are mapped to Disease Ontology and MeSH terms. The associations are classified into three evidence types: Strong experimental evidence, weak experimental evidence and prediction algorithm and miRNAs following the miRBase nomenclature.

PhenomiR [48] provides data from 542 studies which investigate the deregulation of miRNA expression in diseases and biological processes as a systematic, manually curated resource. miRNAs are mapped to miRBase, and diseases are annotated according to the Online Mendelian Inheritance in Man (OMIM) [49] Morbid Map. SM2miR [50] is another useful resource which contains information about research on drugs that affect miRNAs expression levels.

### 4.2. Gene-Based Annotation Resources

Gene Ontology and KEGG are probably the most common databases that store functional annotations used in enrichment analysis. Although, as mentioned previously, Gene Ontology includes miRNAs under their terminology, it is still widely used in indirect miRNA enrichment approaches. Gene Ontology provides a vocabulary for categorizing biological processes, cellular components and molecular functions. Besides the ontology itself, the consortium also provides annotations for several organisms, with evidence-based statements relating a specific gene product to specific ontology terms.

The KEGG database is a very popular and well-established resource that originally focused on metabolic pathways, but currently includes 18 different databases classified into four main categories, namely, systems, genomics, chemicals and health. KEGG is widely known due to its interactive pathways and network maps. In addition to its relevance for basic research, in the last few years, the database began to move towards biomedicine applications integrating human diseases, drugs and other health-related substances.

Being part of the GO consortium, Panther (protein analysis through evolutionary relationships) [51] was born as a classification system of proteins and their genes into families and subfamilies based on their sequence orthology. This classification, along with a tool suite, allows to performs different functional analyses based on Gene Ontology and their inherent pathways annotations. The pathways are complemented with information from Reactome [52], and their own GOSlim version (a subset of the GO ontology with broader terms) is available. While Panther covers several organisms, Reactome only applies to human data and aims to annotate validated information regarding genes, drugs, small molecules, catalysts and regulators throughout more than 1800 pathways organized in a multilevel hierarchical network that could be collapsed into 26 super-pathways, e.g., hemostasis or muscle contraction. Another source of pathway annotation is WikiPathways [53], which was characterized by a crowdsourcing curation and presents more than 2600 pathways. This classification also implements different ways to train new users and ensure quality terms and proper evidence tracking.

Disease to gene databases are also well-established, for example, Online Mendelian Inheritance in Man (OMIM) or Disease Ontology and Human Phenotype Ontology (HPO) [54] are widely used resources. OMIM focuses on human genes and genetic disorders and traits, highlighting the nature of their variations and the resulting phenotypes. Currently, OMIM has over 24,600 entries with approximately 16,000 genes and 8600 phenotypes. The Disease Ontology includes over 9069 disease terms which are interconnected semantically with other databases, such as OMIM. Likewise, the Human Phenotype Ontology provides the most comprehensive normalized vocabulary in order to carry out deep phenotyping in the rare diseases field. Given the heterogeneity of rare diseases, this ontology was adopted by many organizations, i.e., database of genomic variation and phenotype in humans using ensemble resources (DECIPHER) [55] and Orphanet.

Information from these databases can also be used to annotate miRNAs-centered resources by linking them via target genes. For instance, miRPathDB [56] uses Gene Ontology, KEGG, Reactome and WikiPathways.

## 5. Tools for miRNA Functional Enrichment Analysis

In this section, we provide a review of popular tools used for miRNA functional enrichment analysis, focused on annotation sources, available organisms, workflow, bias handling and statistical methods. Table 3 contains a summary of the revised tools specifying the type of annotation, functional analysis method, target gene sources, available annotations and supported organisms. In addition, supplementary material (File S1) contains an overview of results from all the reviewed tools using a list of 26 dysregulated miRNAs in serum exosomes from glioblastoma (GBM) patients [57].

### 5.1. MiRNet

MiRNet [72] offers a unique way of exploring the interactome of miRNAs. Its database includes 11 different miRNA resources and allows the use of custom data. The interactome includes miRNAs and their validated and/or predicted targets, plus, elements like xeno-miRs, transcription factors, epigenetic modifiers, SNPs, pseudogenes, ncRNAs, diseases, and small compounds. All of this information is available for 10 different model organisms. To use the application users can introduce any of the mentioned elements alongside a gene expression data table from mRNA or miRNA experiments. The analysis will report pairwise tables and networks of connections among miRNAs or genes based on all the different elements selected.

From the network, a functional SEA can be applied with the hypergeometric test, including direct and indirect annotations depending on the collection selected. If this is based on genes, an empirical sampling of the test p-values is incorporated along with annotations from GO, KEGG, Reactome or Diseases, meanwhile, if miRNAs are chosen no empirical sampling is applied because direct annotations from TAM are available, allowing over-representation in tissues, diseases, miRNA functions, miRNA clusters, miRNA families and miRNA transcription factors. This dual implementation is motivated by bias in the indirect approach of miRNA enrichment. The empirical sampling follows what is described by Bleazar et al. [18] and the inclusion of direct annotations follows a solution proposed by Godard and van Eyll [19]. The empirical p-values could be re-estimated with another 1000 permutations by resubmitting the functional analysis, but a limitation is that they can only be obtained by using the full set of genes in the network and results are only reported if p-values are below 0.001. If the hypergeometric test is applied, no p-value cut-off exists; therefore, all the related terms are shown. MiRNet is available programmatically via R’s package, web application programming interface (API) and at https://www.mirnet.ca.

### 5.2. GeneCodis

GeneCodis [73] is a popular functional enrichment tool first presented in 2007 [74] as one of the first applications for modular enrichment analysis. It allows enrichment analysis of single annotations, but its main advantage is the extraction of sets of annotations associated with the same set of genes to evaluate statistical significance. It currently supports 15 annotation sources for biological processes, pathways, regulatory elements and drugs. In the last update, GeneCodis incorporated a functionality of miRNAs enrichment analysis via indirect annotation for 5 of 15 available organisms. Target genes with strong evidence were retrieved from the last version of miRTarBase. Additionally, from a list of genes, transcription factors or CpG sites, this database checks which miRNAs are significatively represented.

Depending on the organism selected, different annotations can be used. In both types of enrichment analysis, the significance of the co-annotation or individual term is obtained using either the hypergeometric test or the chi-square tests, and p-values are corrected by false discovery rate (FDR) or using a permutations-based approach. GeneCodis allows users to customize a background set of genes or miRNAs; by default, all annotated genes are considered. Two bias corrections are available, namely, with permutations in concordance with the Blaezard et al. bias solution, and also, in the MEA methodology, a similar grouping of miRNAs targets in co-occurring annotations was proposed by Godard and van Eyll. This tool also implements different visualization capabilities to explore the results beyond standard graphs and tables, such as term clustering that is based on gene sharing by combining principal component analysis and t-SNE (t-distributed Stochastic Neighbor Embedding). GeneCodis is available at https://genecodis.genyo.es.

### 5.3. MiEAA

The miRNA Enrichment Analysis and Annotation tool (miEAA) [75] implements GSEA and SEA for miRNAs. It consists of a comprehensive database of more than 40 different collections obtained with direct and indirect annotations of miRNAs. The main sources are 15 databases that allow users to explore associations with pathways, diseases, miRNA nature and classification, drugs, functions, cells and tissues, targets, and transcription factors. To avoid bias in miRNA enrichment analysis, a majority of sources providing direct annotations were collected. The statistical method used in the SEA is the Fisher’s exact test, whereas, GSEA uses an un-weighted variant of the algorithm which corresponds to a Kolmogorow-Smirnow test. The static GSEA running sum plots shows a simulated background distribution computed by randomly permuting the input list 100 times and traversing the running sum for each random permutation. Furthermore, six different procedures are available for multiple tests correction.

MiEAA uses a list of precursors or mature miRNAs as input, where the user can select from the enrichment methodologies. In the case of SEA, a background set can be uploaded, otherwise, all annotated miRNAs/precursors are used as a reference. To perform GSEA the input must be sorted by some criterion, such as fold change. Once submitted, the results gather all the categories into a single table. For the top 100, sorted by p-value, a word cloud and heatmaps of miRNA versus annotation are created, whereas interactive enrichment graphs are shown for GSEA. MiEAA is available programmatically via API and at: https://ccb-compute2.cs.uni-saarland.de/mieaa2.

### 5.4. MIENTURNET

MIENTURNET (miRNA enrichment turned network) [76] was published as a tool to study miRNA-target interactome. Every miRNA and target gene is extracted from TargetScan and those experimentally validated from miRTarBase. The six organisms shared between these databases are included in the tool. Four sources of annotations available for functional analysis performance, specifically, KEGG, Reactome, WikiPathways, and, for human data only, Disease Ontology. These are linked to genes, indicating that over-representation analysis is performed over the miRNAs targets and designating it as an indirect annotation approach.

As input, a list of genes or miRNAs can be used. In both cases, this will trigger an enrichment analysis of miRNAs or targets, respectively, by querying, TargetScan and miRTarBase simultaneously, which in turn provide two interactomes of miRNA-gene pairs, one is based on predicted interactions and the other on validated interactions. Targets can be filtered by the type of evidence for miRTarBase and two prediction scores, the cumulative weighted context++ and the probability of conserved targeting. Functional analysis can be performed using all the targets of up to 10 miRNAs from the interactomes. The significance of terms is addressed with the hypergeometric test, and p-values are corrected using Benjamini-Hochberg FDR. As a background, by default, the whole genome is used; otherwise, the input genes are used. MIENTURNET is available at http://userver.bio.uniroma1.it/apps/mienturnet.

### 5.5. TAM

TAM [58] is a dedicated human miRNA functional analysis tool whereby, through manual curation of more than 9000 papers, a database was created to characterize around 1200 distinct miRNAs. These are associated in a total of 1238 miRNA sets distributed throughout 547 diseases, 158 biological functions, 166 transcription factors, 6 tissues and 211 clusters and 151 families of miRNAs. Interestingly, the nomenclatures of disease and functional terms were normalized regarding other well-established ontologies (ICD-10-CM, Disease Ontology, MeSH, OMIM, HPO and Gene Ontology). Another unique feature of this tool is that for almost all miRNA-disease associations, miRNAs are classified into two groups, namely, (1) up-miRNA, i.e., miRNAs that are up-regulated in disease conditions, or exhibit disease-promoting function according to the phenotype from gene permutation assays, and conversely, (2) down-miRNA, curated in the same way.

TAM is an example of miRNA functional analysis using exclusively direct annotations. Taking into consideration the bias regarding the knowledge of miRNAs separate from the direct annotations, this tool provides an option to mask cancer and non-standard miRNA terms when performing analyses. TAM offers two types of enrichment processes, a SEA with the hypergeometric test, and notably, a comparison of query miRNAs signature (up and down-regulated) with the disease-based signatures stored in the database. TAM is available at http://www.lirmed.com/tam2.

### 5.6. MiTALOS

MiTALOS [77] is a dedicated functional analysis tool for miRNA in which Preusse et al. integrated three key aspects. First, from CLIP-seq studies of StarBase [78], they extracted high-quality pairs miRNA-target, but also considered predicted pairs from TargetScan and miRanda [79]. Second, they captured tissue-specific gene expression from the latest version of EBI Expression Atlas. Lastly, they included three major pathway databases: KEGG, WikiPathways and Reactome. As a result, this tool incorporates the specificity of expression signatures of miRNAs and target transcripts in different tissues to improve the functional analysis of miRNAs. All miRNA information is relative to *H. sapiens* and *M. musculus*.

The approach of miTALOS is an indirect annotation, but thanks to a dynamic database tissue filter, the results provide insight and may overcome the described bias. In detail, they establish that the expression of miRNAs and pathways is tissue-specific, therefore miRNAs, genes and pathways can be discarded in the face of a functional analysis if they are not expressed in the selected tissue. Terms significance is obtained via Fisher’s exact test and corrected using the Benjamini-Hochberg procedure. MiTALOS is available at http://mips.helmholtz-muenchen.de/mitalos.

### 5.7. MiRSystem

MiRSystem [80] is a dedicated source to perform miRNA functional enrichment analysis. Its miRNAs database is built from miRBase and allows two species, *H. sapiens* and *M. musculus.* Regarding the functional annotations, five databases are collected: KEGG, Gene Ontology, BioCarta, Pathway Interaction Database, and Reactome.

This tool uses the indirect annotation approach and miRNAs are transformed to target genes by combining different prediction algorithms and two experimentally validated databases. A list of targets is obtained after prediction by a minimum number of algorithms. By default, the experimentally validated targets are added. The test incorporated is the hypergeometric test which is complemented by calculating the observed/expected (O/E) ratio (i.e., observed genes divided by expected genes under a term) and an empirical p-value using a permutations test. Similar to other tools, the calculation of an empirical p-value is a method to overcome bias. A weighted enrichment analysis also was developed, which is a unique method compared to the other tools. If miRNAs are introduced along with a numeric value, e.g., the expression, the enriched pathways are scored according to the expression of the associated miRNAs. The weight for one miRNA is calculated by dividing its absolute expression value by the absolute sum of the expression values of all input miRNAs. Thereafter, the pathway ranking score is obtained by totaling the weight of its miRNA times its enrichment 2log (*p*-value). MiRSystem is available at http://mirsystem.cgm.ntu.edu.tw.

### 5.8. DIANA miRPath

miRPath [81] is part of the DIANA framework and links miRNAs to Gene Ontology and KEGG. miRNAs are converted to predicted targets by TargetScan or DIANA microT-CDS and/or experimentally validated targets stored in DIANA TarBase. These associations are available for seven model organisms.

Before performing enrichment analysis for each introduced miRNA, its predicted targets can be filtered according to each algorithm’s own score system and then collapsed into a single list either by the intersection of a minimum number of associated miRNAs or by the union of all targets. miRPath fits into the indirect annotation category, but it comes with statistical implementations for bias assessment. To obtain p-values, miRPath can use Fisher’s exact test and the DAVID’s EASE score [82] optionally, p-values can be corrected via Benjamini-Hochberg’s FDR. These values undergo permutation testing derived from an adaptation of the sampling algorithm presented by Blaezard et al., resulting in empirical p-value. Finally, a method consisting of a meta-analysis statistic was developed to enable the identification of pathways controlled by multiple miRNAs by examining each one individually and subsequently combining the result probabilities and test statistics. Additionally, functional analysis can be performed in the opposite direction, i.e., in the reverse search module, to discover over-representation of miRNAs from a set of targets of a single GO term or KEGG pathway. DIANA miRPath is available at: http://snf-515788.vm.okeanos.grnet.gr.

## 6. Conclusions and Discussion

There are numerous databases for miRNAs that provide various types of data, including predicted and experimental evidence of miRNA and target gene associations. However, the availability of miRNAs functional information gained importance over the last few years as an essential step to interpret high-throughput experiments and decipher the biological processes in which they are involved. In this context, several miRNA enrichment analysis tools were recently introduced, and are becoming very useful resources in miRNA research. These tools can combine both miRNA target genes plus gene-based annotations databases and/or direct miRNA functional annotations databases.

Initially, miRNAs functional analysis methods were based on SEA using target genes information, but different efforts are being realized to provide high-quality direct annotations for miRNAs. In the indirect approach, it is important to use well-validated target genes, while the use of direct annotations, miRNA-based databases, requires a well-established and normalized vocabulary. In order to decipher specific pathways and functions, it is essential the specificity of target prediction algorithms. Therefore, target validation information or integration of other data sources, such as gene expression, is very convenient in functional analysis. Tools like GeneCodis, use uniquely target genes validated by miRTarBase offering MEA, but some others stand out because they incorporate direct annotations, such as TAM. Differently, miEEA implements GSEA and both types of annotations. These varying implementations, in addition to statistical tests and supported organisms, offer several alternatives to perform miRNA enrichment analysis. It is known that each study requires a different setup, whatsoever, motivated by the bias in the indirect approach, tools that have miRNAs direct annotations should be preferred whenever possible. Nonetheless, it could be argued that direct annotations are still lacking and are not widely implemented in miRNAs functional analysis tools. Thus, if miRNAs direct annotations do not meet the research specificity level and gene-based annotations are necessary, the bias handling method in the indirect workflow must be noticed. Empirical sampling is the most common method to assess the bias, while the clustering of annotations in GeneCodis and the background specificity of a selected tissue in tools like MiTalos are less implemented. In the case of TAM, although it uses direct annotations, an option to mask cancer and unspecific terms are available when the miRNAs list under study is not related to the pathology.

This review provided an overview of the most widely used resources for miRNA functional analysis, remarking upon the main features in terms of the type of annotation, statistical test, organism or enrichment analysis method. We expect this review to be useful in selecting the most appropriate resource depending on the experimental context.

## Figures and Tables

**Table 1:** Summary of the miRNA target prediction algorithms described.

Tool	Learn Attributes Remark	Organisms	URL	Last Up-Date
TarPmiR	Novel features from CLASH data	*Homo sapiens*	http://hulab.ucf.edu/research/projects/miRNA/TarPmiR/	2016
TargetScan	Score for mammal predictions	*H. sapiens, Mus musculus, Rattus norvegicus, Pan troglodytes, Macaca mulatta, Canis familiaris, M. domestica, Bos taurus, C. elegans, Drosophila melanogaster, Danio rerio, Gallus gallus, Xenopus tropicalis*	http://www.targetscan.org	2015
MiRTarget	Functional targets from RNA-seq	H. sapiens, M. musculus, R. norvegicus, C. familiaris, G. gallus	http://mirdb.org/	2019
DIANA microT-CDS	PAR-CLIP data, targets in CDS and 3′ UTR	*H. sapiens, M. musculus, C. elegans, D. melanogaster*	http://diana.imis.athena-innovation.gr/DianaTools/index.php?r=microT_CDS	2013

**Table 2:** Summary of the reviewed databases with experimentally validated miRNA targets genes.

Tool	Curation	Target-miRNA	Organisms	URL	Last Update
miRTarBase	11,021 articles, 331 CLIP-seq datasets	479,340	32	http://mirtarbase.cuhk.edu.cn	2020
DIANA-TarBase	1208 articles, 353 datasets, 34 methods	665,843	18	https://carolina.imis.athena-innovation.gr/diana_tools/web/index.php?r=tarbasev8	2017

**Table 3:** Summary of the revised miRNA functional enrichment analysis tools.

Tool	Annotation/Bias Handling	Method	Targets	Sources of Annotations	Organism
miRNet	Indirect, Direct/Empirical sampling	SEA	Validated, predicted	GO, KEGG, Reactome, TAM [58]	*H. sapiens, M. musculus, R. norvegicus, B. taurus, Sus scrofa, G. gallus, D. melanogaster, C. elegans, D. rerio, Schistosoma mansoni*
GeneCodis	Indirect/Empirical sampling, co-annotation	MEA, SEA	Validated	DoRothEA [59], miRTarBase, GO, KEGG Pathways, MGI [60], Panther, Reactome, WikiPathways, CTD [61], HPO, LINCS [62], OMIM, PharmGKB [63]	*D. melanogaster, D. rerio, H. sapiens, M. musculus, R. norvegicus.*
miEAA	Indirect, Direct/None	SEA, GSEA	Validated, redicted	GO, HMDD, KEGG, miRandola [64], miRBase, miRPathDB, miRTarBase, miRWalk [65], MNDR, NPInter [66], RNALocate [67], SM2miR, TAM, TissueAtlas [68], TransmiR [69], Literature	*H. sapiens, M. musculus. R. norvegicus, Arabidopsis thaliana, B. taurus, C. elegans, D. melanogaster, D. rerio, G. gallus, S. scrofa*
MIENTURNET	Indirect/None	SEA	Validated, predicted	KEGG, Reactome, WikiPathways, Disease Ontology	*H sapiens, M. musculus, R. norvegicus, C. elegans, D. melanogaster, D. rerio*
TAM	Direct/Mask cancer and unspecific terms	SEA	-	Literature	*H. sapiens*
miTALOS	Indirect/Background specificity	SEA	Validated, predicted	KEGG, WikiPathways, Reactome	*H. sapiens*, *M. musculus*
miRSystem	Indirect/Empirical sampling	SEA	Validated, predicted	KEGG, GO, BioCarta [70], Pathway Interaction Database [71], Reactome.	*H. sapiens, M. musculus*
DIANA miRPath	Indirect/Empirical sampling	SEA	Validated, predicted	GO, KEGG	*H. sapiens, M. musculus, R. norvegicus, D. melanogaster, C. elegans, G. gallus, D. rerio*.

## Supplementary Materials

Available online at https://www.mdpi.com/2218-273X/10/9/1252/s1, File S1: Tables S1–S9. Tables S1–S8: Results from each tool for a 26 miRNAs signature in glioblastoma patients. Table S9: Table with tools and URLs not included from mentioned resources.
