# Supplementary material for: Computational Methods and Software Tools for Functional Analysis of miRNA Data
Source: Biomolecules. 2020 Aug 28;10(9):1252. doi: 10.3390/biom10091252 (PMC7563698; doi:10.3390/biom10091252)
Supplement: Supplementary file 1 [file biomolecules-10-01252-s001.pdf]

biomolecules

Supplementary material:

Computational methods and software tools for functional analysis of miRNA data

Adrian Garcia-Moreno[1], Pedro Carmona-Saez [1, 2]

[1] Bioinformatics Unit, GENYO Centre for Genomics and Oncological Research: Pfizer/University of Granada/Andalusian Regional Government, PTS Granada, 18016 Granada, Spain

[2] Department of Statistics, University of Granada, 18071 Granada, Spain.

Table S1. miRNet top 25 KEGG enriched terms, using validated targets of TarBase and MirTarBase.

Name	Pval	adj.Pval
Pathways in cancer	7.22E-12	7.22E-10
Protein processing in endoplasmic reticulum	1.04E-10	5.2E-09
RNA transport	1.38E-09	4.6E-08
Neurotrophin signaling pathway	5.27E-09	1.3175E-07
Cell cycle	1.05E-08	2.1E-07
Prostate cancer	3.97E-08	6.616667E-07
Renal cell carcinoma	1.02E-07	1.457143E-06
p53 signaling pathway	1.37E-07	1.7125E-06
Influenza A	5.63E-07	6.090909E-06
Chagas disease (American trypanosomiasis)	6.1E-07	6.090909E-06
Chronic myeloid leukemia	6.7E-07	6.090909E-06
Epstein-Barr virus infection	7.85E-07	6.276923E-06
HTLV-I infection	8.16E-07	6.276923E-06
Small cell lung cancer	3.3E-06	2.357143E-05
mRNA surveillance pathway	4.21E-06	2.70625E-05
Pancreatic cancer	4.33E-06	2.70625E-05
Colorectal cancer	7.22E-06	4.247059E-05
ErbB signaling pathway	1.22E-05	6.777778E-05
Bacterial invasion of epithelial cells	1.32E-05	6.947368E-05
Focal adhesion	1.87E-05	9.35E-05
Regulation of actin cytoskeleton	3.59E-05	0.0001709524
Toxoplasmosis	5.23E-05	0.0002377273
Wnt signaling pathway	6.03E-05	0.0002621739
Fc gamma R-mediated phagocytosis	6.96E-05	0.00029
MAPK signaling pathway	7.7E-05	0.0002951852

Table S2. GeneCodis top 25 results of co-annotated KEGG enriched terms.

**term**	**hyp_pval**	**hyp_pval_adj**
Pathways in cancer	1.84293E-10	4.75886E-08
Pathways in cancer,Human papillomavirus infection,Pancreatic cancer	1.72536E-10	5.01217E-08
Human cytomegalovirus infection,Pathways in cancer,Hepatitis C,Human papillomavirus infection,Pancreatic cancer	1.59675E-10	5.30122E-08
Human cytomegalovirus infection,Pathways in cancer,Melanoma,Breast cancer,Hepatitis C,Human papillomavirus infection,Non-small cell lung cancer,Hepatocellular carcinoma,Pancreatic cancer,**Glioma**	2.49424E-10	5.79661E-08
Human papillomavirus infection	5.19338E-11	6.03471E-08
Pathways in cancer,Endocrine resistance	1.58998E-10	6.15851E-08
Pathways in cancer,Breast cancer,PI3K-Akt signaling pathway,Non-small cell lung cancer,Pancreatic cancer	8.62528E-11	6.68171E-08
Human cytomegalovirus infection,Kaposi sarcoma-associated herpesvirus infection,Pathways in cancer,Hepatitis C,Human papillomavirus infection,Cellular senescence,Chronic myeloid leukemia,Pancreatic cancer	1.49007E-10	6.92583E-08
Pathways in cancer,Human papillomavirus infection,Cellular senescence	1.20916E-10	7.02524E-08
Human papillomavirus infection,Cellular senescence	4.14997E-11	9.64453E-08
Human cytomegalovirus infection,Pathways in cancer,Melanoma,Breast cancer,Hepatitis C,Human papillomavirus infection,Non-small cell lung cancer,Hepatocellular carcinoma,Cushing syndrome,Pancreatic cancer,**Glioma**	1.10738E-09	1.07231E-07
Pathways in cancer,Cellular senescence,Chronic myeloid leukemia,Pancreatic cancer	1.08208E-09	1.09337E-07
Pathways in cancer,Breast cancer,Non-small cell lung cancer,Pancreatic cancer	1.08208E-09	1.09337E-07
Pathways in cancer,Human papillomavirus infection,FoxO signaling pathway	1.08208E-09	1.09337E-07
Human cytomegalovirus infection,Kaposi sarcoma-associated herpesvirus infection,Human papillomavirus infection,Cellular senescence	5.26533E-10	1.11242E-07
Pathways in cancer,PI3K-Akt signaling pathway,Endocrine resistance	1.52138E-09	1.17856E-07
Human papillomavirus infection,AGE-RAGE signaling pathway in diabetic complications	1.52138E-09	1.17856E-07
Pathways in cancer,Breast cancer,Non-small cell lung cancer,Endocrine resistance,Pancreatic cancer	9.98868E-10	1.22177E-07
Pathways in cancer,Cellular senescence	8.99431E-10	1.22958E-07
Pathways in cancer,Pancreatic cancer	1.07151E-09	1.24509E-07
Human cytomegalovirus infection,Pathways in cancer,PI3K-Akt signaling pathway,Hepatitis C,Human papillomavirus infection,Pancreatic cancer	6.44082E-10	1.24737E-07
Pathways in cancer,Gastric cancer,PI3K-Akt signaling pathway,Endocrine resistance,Prostate cancer	1.51721E-09	1.25929E-07
Pathways in cancer,Breast cancer,Endocrine resistance	9.83365E-10	1.26963E-07
PI3K-Akt signaling pathway,Human papillomavirus infection	1.69894E-09	1.27366E-07
Human cytomegalovirus infection,Pathways in cancer,Melanoma,Breast cancer,PI3K-Akt signaling pathway,Hepatitis C,Human papillomavirus infection,Non-small cell lung cancer,Hepatocellular carcinoma,Pancreatic cancer,**Glioma**	8.81732E-10	1.28072E-07

Table S3. MiEAA top 25 KEGG enriched terms.

Subcategory	P-value	P-adjusted
Chagas disease American trypanosomiasis	3.13E-05	0.0050175
Glucagon signaling pathway	4.65E-05	0.0050175
Th1 and Th2 cell differentiation	3.75E-05	0.0050175
Th17 cell differentiation	7.24E-05	0.0058637
Carbohydrate digestion and absorption	0.000219	0.0141631
AMPK signaling pathway	0.000403	0.0217674
GnRH secretion	0.000867	0.028694
Huntington disease	0.000886	0.028694
Inflammatory bowel disease IBD	0.000735	0.028694
Thyroid hormone signaling pathway	0.000812	0.028694
Allograft rejection	0.0011694	0.031573
Non-alcoholic fatty liver disease NAFLD	0.0011209	0.031573
Acute myeloid leukemia	0.0015098	0.0349415
Autoimmune thyroid disease	0.0014424	0.0349415
Antigen processing and presentation	0.0017465	0.035367
Type II diabetes mellitus	0.0016736	0.035367
Aldosterone-regulated sodium reabsorption	0.0020635	0.0393281
Amoebiasis	0.0026507	0.0442445
Cholinergic synapse	0.0025683	0.0442445
Glycosaminoglycan biosynthesis - chondroitin sulfate dermatan sulfate	0.0027311	0.0442445
Phosphatidylinositol signaling system	0.0033911	0.0523206
Adherens junction	0.0051526	0.0536
Arrhythmogenic right ventricular cardiomyopathy ARVC	0.0046519	0.0536
Ascorbate and aldarate metabolism	0.0046191	0.0536
Bacterial invasion of epithelial cells	0.0042319	0.0536

Table S4. MIENTURNET top 25 results KEGG enriched terms of each miRNA targets individually.

microRNA	Description	p-value	FDR
hsa-miR-340-5p	Melanoma	2.59967491035388E-12	1.0125049650852E-10
hsa-miR-340-5p	Viral carcinogenesis	6.74257839364133E-11	1.31302842402489E-09
hsa-miR-340-5p	FoxO signaling pathway	1.90666260320168E-10	2.4753163620513E-09
hsa-miR-340-5p	Non-small cell lung cancer	2.57821095097297E-10	2.51036329436842E-09
hsa-miR-340-5p	MicroRNAs in cancer	1.91677810257791E-09	1.49306925885016E-08
hsa-miR-340-5p	Epstein-Barr virus infection	4.02659444750818E-09	2.48363138790062E-08
hsa-miR-340-5p	Proteoglycans in cancer	4.46382398095652E-09	2.48363138790062E-08
hsa-miR-340-5p	Human cytomegalovirus infection	8.54900296120934E-09	4.16201459953613E-08
hsa-miR-340-5p	Prolactin signaling pathway	3.25158438545421E-08	1.40711838902697E-07
hsa-miR-340-5p	Cellular senescence	4.0415912817941E-08	1.48052458901918E-07
hsa-miR-340-5p	**Glioma**	4.62000631964526E-08	1.48052458901918E-07
hsa-miR-340-5p	Pancreatic cancer	4.94175099307752E-08	1.48052458901918E-07
hsa-miR-340-5p	Chronic myeloid leukemia	4.94175099307752E-08	1.48052458901918E-07
hsa-miR-182-5p	Prostate cancer	3.00495649156945E-09	1.80297389494167E-07
hsa-miR-340-5p	PI3K-Akt signaling pathway	1.96463654727908E-07	5.46553024431023E-07
hsa-miR-21-5p	MicroRNAs in cancer	1.37950127101587E-08	1.23429061090894E-06
hsa-miR-340-5p	Cell cycle	5.81468582370863E-07	1.50977807352435E-06
hsa-miR-486-5p	Cellular senescence	5.22238532154296E-08	2.07477530143772E-06
hsa-miR-486-5p	Hepatocellular carcinoma	7.58090975525323E-08	2.07477530143772E-06
hsa-miR-21-5p	FoxO signaling pathway	4.91218546675311E-08	2.19755665617902E-06
hsa-miR-340-5p	Measles	1.0270294043123E-06	2.50000578681283E-06
hsa-miR-21-5p	Signaling pathways regulating pluripotency of stem cells	1.05171841857678E-07	3.13670405540443E-06
hsa-miR-486-5p	Melanoma	2.01894144563342E-07	3.26108890412478E-06
hsa-miR-486-5p	**Glioma**	2.38310342993734E-07	3.26108890412478E-06
hsa-miR-21-5p	Proteoglycans in cancer	2.09943511723231E-07	3.9282064398909E-06

Table S5. TAM top 25 results where KEGG is not available due to TAM using its own annotations.

Term	P-value	FDR
Carcinoma, Gastric	5.67E-08	6.87E-05
Carcinoma, Breast	1.3E-07	7.88E-05
Breast Neoplasms	6.22E-07	0.000251
Carcinoma, Prostate	1.59E-06	0.00048
Osteosarcoma	3.52E-06	0.000852
Carcinoma, Colon	4.35E-06	0.000878
Prostate Neoplasms	5.12E-06	0.000886
Atrial Fibrillation	8.96E-06	0.00136
Melanoma	1.25E-05	0.00168
Carcinoma, Basal Cell	2.31E-05	0.0028
**Glioma**	3.4E-05	0.00374
Carcinoma, Thyroid	4.11E-05	0.00414
Carcinoma, Lung	4.44E-05	0.00414
Coronary Heart Diseases	5.81E-05	0.0044
Adenocarcinoma, Lung	6.25E-05	0.00445
Adenocarcinoma, Gastric	5.67E-05	0.00458
Carcinoma, Hepatocellular	5.37E-05	0.00465
Gastrointestinal Neoplasms	9.51E-05	0.00606
hsa-mir-379 cluster	0.000102	0.00619
Ovarian Neoplasms	9.25E-05	0.00623
Lung Neoplasms	0.000125	0.00719
Acute Myocardial Infarction	0.000173	0.00912
Polycystic Ovarian Syndrome	0.000171	0.00939
Leukemia	0.000189	0.00956
Early-Stage Non-Small-Cell Lung Carcinoma	0.000228	0.011

Table S6. MiTALOS only 12 terms shown because a 0.05 significance cut-off is imposed. Also results from Wikipathways (wp) are shown.

Source	Name	p-value
wp	Mesodermal Commitment Pathway	0.0006362389
wp	Endoderm Differentiation	0.0028072615
wp	BMP Signalling and Regulation	0.0049942647
wp	Neural Crest Differentiation	0.0051841802
wp	BDNF signaling pathway	0.0082582503
kegg	Circadian rhythm	0.0128012213
kegg	Axon guidance	0.0141600339
kegg	TGF-beta signaling pathway	0.0269067405
wp	Ectoderm Differentiation	0.0281909391
wp	Regulation of Microtubule Cytoskeleton	0.0426678613
kegg	Circadian entrainment	0.0443499462
wp	Interactome of polycomb repressive complex 2 (PRC2)	0.0466741508

Table S7. MiRSystem only 11 terms shown because a 0.05 significance cut-off is imposed.

CATEGORY	TERM	RAW_P_VALUE	EMPIRICAL_P_VALUE
PATHWAY_INTERACTION_DATABASE	PDGFR-BETA_SIGNALING_PATHWAY	5.44E-02	1.12E-01
KEGG	COLORECTAL_CANCER	2.75E-02	1.63E-01
KEGG	VASCULAR_SMOOTH_MUSCLE_CONTRACTION	5.44E-02	1.66E-01
PATHWAY_INTERACTION_DATABASE	SIGNALING_EVENTS_MEDIATED_BY_HDAC_CLASS_III	1.74E-02	1.68E-01
REACTOME	METABOLISM_OF_AMINO_ACIDS_AND_DERIVATIVES	7.36E-02	2.03E-01
REACTOME	MUSCLE_CONTRACTION	2.18E-02	2.42E-01
KEGG	PATHWAYS_IN_CANCER	1.29E-01	2.58E-01
KEGG	ARGININE_AND_PROLINE_METABOLISM	2.40E-02	2.78E-01
PATHWAY_INTERACTION_DATABASE	ATM_PATHWAY	1.52E-02	2.98E-01
KEGG	SYSTEMIC_LUPUS_ERYTHEMATOSUS	5.85E-02	3.53E-01
REACTOME	DNA_REPAIR	4.70E-02	3.88E-01

Table S8. DIANA miRPath top 25 terms enrichmed of targets obtained with microT-CDS

KEGG pathway	p-value
Proteoglycans in cancer	4.24754302777E-12
GABAergic synapse	7.29751476932E-07
**Glioma**	7.29751476932E-07
Signaling pathways regulating pluripotency of stem cells	3.96577193346E-06
ErbB signaling pathway	9.43103702335E-06
Pathways in cancer	9.43103702335E-06
Prion diseases	1.15236814217E-05
TGF-beta signaling pathway	1.9250366962E-05
Adherens junction	1.9250366962E-05
Prostate cancer	2.40147596687E-05
Wnt signaling pathway	5.09311778067E-05
Neurotrophin signaling pathway	5.29898631152E-05
Renal cell carcinoma	8.21252953281E-05
Transcriptional misregulation in cancer	8.82224240435E-05
FoxO signaling pathway	0.000108567215874
Hippo signaling pathway	0.000128403815899
Thyroid hormone signaling pathway	0.000128403815899
Axon guidance	0.000141614237881
T cell receptor signaling pathway	0.000143100790325
Glutamatergic synapse	0.000310995879636
Pancreatic cancer	0.000354922474548
Ras signaling pathway	0.00038388961646
Focal adhesion	0.000557734213272
Choline metabolism in cancer	0.000585313623452
Morphine addiction	0.000755390427698

Table S9. Web resources referenced in the main text whose URL has not been included are gathered in the following table.

Resource	Type	URL
miRCarta	miRNA database	https://mircarta.cs.uni-saarland.de
mirGeneDB	miRNA database	http://mirgenedb.org
miRBase	miRNA database	http://www.mirbase.org
miRMaster	miRNA-seq analytic	https://ccb-compute.cs.uni-saarland.de/mirmaster

miRCancer	miRNA annotations database	http://mircancer.ecu.edu
miR2Disease	miRNA annotations database	http://www.mir2disease.org
Human miRNA Disease Database (HMDD)	miRNA annotations database	http://www.cuilab.cn/hmdd
Mammal NcRNA-Disease Repository (MNDR)	miRNA annotations database	http://www.rna-society.org/mndr
Plant miRNA Encyclopedia (PmiREN)	miRNA annotations database	http://www.pmiren.com
PhenomiR	miRNA annotations database	http://mips.helmholtz-muenchen.de/phenomiR
SM2miR	miRNA annotations database	http://www.jianglab.cn/SM2miR/
EpimiR	miRNA annotations database	http://bioinfo.hrbmu.edu.cn/EpimiR
miRPathDB	miRNA annotations database	https://mpd.bioinf.uni-sb.de
Gene Ontology (GO)	gene annotations database	http://geneontology.org
Kyoto Encyclopedia of Genes and Genomes (KEGG)	gene annotations database	https://www.genome.jp/kegg
Panther (Protein ANalysis THrough Evolutionary Relationships)	gene annotations database	http://www.pantherdb.org
WikiPathways	gene annotations database	https://www.wikipathways.org
Online Mendelian Inheritance in Man (OMIM)	gene annotations database	https://www.omim.org
Human Phenotype Ontology (HPO)	gene annotations database	https://hpo.jax.org
Disease Ontology	gene annotations database	https://disease-ontology.org
Reactome	gene annotations database	https://reactome.org
Orphanet	drug and rare disease annotations database	https://www.orpha.net
DECIPHER	Omics database	https://decipher.sanger.ac.uk
The Cancer Genome Atlas (TCGA)	Cancer omics database	https://www.cancer.gov/about-nci/organization/ccg/research/structural-genomics/tcga
NCBI-Sequence Read Archive (SRA)	Omics database	https://www.ncbi.nlm.nih.gov/sra

© 2020 by the authors. Submitted for possible open access publication under the terms and conditions of the Creative Commons Attribution (CC BY) license (http://creativecommons.org/licenses/by/4.0/).
